# Prevalence of overweight in children with bone fractures: a case control study

**DOI:** 10.1186/1471-2431-12-166

**Published:** 2012-10-22

**Authors:** Giuliana Valerio, Francesca Gallè, Caterina Mancusi, Valeria Di Onofrio, Pasquale Guida, Antonino Tramontano, Edoardo Ruotolo, Giorgio Liguori

**Affiliations:** 1 Department of Studies of Institutions and Territorial Systems School of Movement Sciences (DiSIST), Parthenope University, Via Medina 40, Naples, 80133, Italy; 2 Unit of Orthopaedics and Traumatology, AORN Santobono-Pausilipon Children’s Hospital, Naples, Italy; 3 Department of Pediatric Surgery, AORN Santobono-Pausilipon Children’s Hospital, Naples, Italy; 4 Medical Direction, AORN Santobono-Pausilipon Children’s Hospital, Naples, Italy

**Keywords:** Fractures, Gender, Inactivity, Lifestyle, Overweight

## Abstract

**Background:**

Children's fractures have been enlisted among orthopaedics complaints of childhood obesity. Unhealthy lifestyle behaviours may contribute to increased risk. This study described the prevalence of overweight/obesity in children and adolescents reporting a recent fracture in relation to gender, dynamic of trauma, and site of fracture.

**Methods:**

Four-hundred-forty-nine children and adolescents with fracture and 130 fracture-free controls were recruited from a large children’s hospital. The interaction between overweight and gender, dynamic of trauma, site of fracture was explored. Sports participation, television viewing, and calcium intake were also investigated.

**Results:**

Overweight/obesity rate was increased in girls with fracture either at the upper or the lower limb (p= 0.004), while it was increased only in boys with fracture at the lower limb (p <0.02). Overweight/obesity rate did not differ between groups with low or moderate trauma. TV viewing ≥ 2 hrs was more frequent in children with fractures than controls (61.5% vs 34.5%, p =0.015) in the overweight/obese group.

**Conclusions:**

The increased prevalence of overweight/obesity in children with fractures is related to gender and site of fracture. Higher levels of sedentary behaviours characterize overweight children reporting fractures.

## Background

Children's fractures represent a common injury during childhood and adolescence. Several studies have shown the role of numerous factors in determining children fractures, such as low bone mass and bone mineral density, low calcium intake, consumption of carbonated beverages, use of drugs [1-4]. In contrast to the protective effect of obesity on fracture rates in adults [5], the current literature demonstrates an increased risk for the occurrence of fractures in overweight children [6,7]. The prevalence of childhood obesity has more than doubled in the last decades in many regions of the world and it is now recognized as a major medical and public health problem. Great concern is turned to the skeletal conditions of obese children, in terms of disability, health-related quality of life, and health-care costs. Complications of fractures are amplified in term of increased surgical times, increased risk of wound infections, and increased time to ambulation in obese subjects [8].

Obesity in children is associated with unhealthy nutrition, inactivity and low physical fitness, that may contribute to increased risk of fractures. While it is well known that childhood fractures are more common in boys than girls and that upper limb fractures prevail on lower limb fractures [9], there are no data on possible interactions between overweight status and gender, dynamics or site of fracture.

The knowledge of the epidemiology of fractures is fundamental to choose the adequate prevention and control strategies for the target population. Therefore the aim of this study was to describe the prevalence of overweight/obesity in children and adolescents with fractures in relation to gender, dynamic of trauma, and site of fracture. The role of sports participation, inactivity, and calcium assumption was also investigated.

## Methods

This was a case–control study conducted on 579 children at the Santobono-Pausilipon Children’s Hospital in Naples (southern Italy). This hospital is the largest children’s hospital in the Campania region, providing inpatient and outpatient services in emergency and trauma medicine in children < 14 years of age within the metropolitan area of Naples. The study commenced on 1 January 2008 and was terminated on 30 June 2008. In 2008, the metropolitan area included 4,434,000 residents, of whom 17.3% (767,082 subjects) were < 14 years of age. The investigation was approved by the Institutional board of the Hospital and written informed consent was obtained from all participants and/or their parents or legal guardians in accordance with the revised version of the Helsinki Declaration regarding research involving human subjects.

Cases (n = 449) were children consecutively admitted for a recent fracture to the outpatient clinic of the Department of Orthopaedics and Traumatology. The inclusion criteria were age ≥ 2 years, residence in the Campania region, trauma due to accidental causes. The exclusion criteria were fractures due to severe trauma, presence of any specific pathologic process known to affect bone and mineral metabolism, presence of any specific treatment known to affect bone and mineral metabolism. Controls (n = 130) were children consecutively followed at the outpatient Department of Surgery of the same hospital for minor surgery complaints (removal of skin lesions, in growing toe-nails, lymphadenopathy, dermoid cyst, appendicitis, genitourinary surgery, hernia repairs). Controls were age and sex-matched with cases (Table 1). Exclusion criterion for controls was having reported a fracture in the present and in the past history.

**Table 1:** Demographic characteristics of cases and controls

	**Cases**	**Controls**
**Number**	449	130
**Gender (M/F) (%)**	292/157 (65/35)	83/47 (65/35)
**Age (yrs)**	8.7±2.9	8.3±2.9
Height (cm)*	135.4±19.3	130.9±18.8
Height-SDS***	0.8±1.2	0.4±1.2
Weight (kg)*	39.4±15.0	35.5±17.4
**BMI (kg/m**^**2**^)*	20.5±4.5	19.4±4.9
BMI-SDS **	1.35 (−5; 5)	0.98 (−3; 3)
**Father’s educational level n (%)**^**a**^		
**Elementary**	35 (8.6)	17 (13.4)
**Middle school**	177 (43.7)	63 (49.6)
**High school**	159 (39.2)	42 (33.0)
**Degree**	34 (8.4)	5 (3.9)
**Mother’s educational level n (%)**^**a**^		
**Elementary**	38 (9.3)	19 (14.7)
**Middle school**	171 (42.0)	53 (41.0)
**High school**	164 (40.2)	50 (38.7)
**Degree**	34 (8.3)	7 (5.4)

Data are expressed as mean±SD or number (%), as appropriate.

Variables not normally distributed (BMI-SDS) were logarithmically transformed; results are expressed as untransformed values (median, min; max). The independent sample *t*-test was used to compare the means of continuous variables, while the chi-square test was used for categorical variables.

* p < 0.01 ** p = 0.008 *** p < 0.001.

^**a**^ Information about parents educational level was not available for all the cases and controls.

Anthropometric measurements were carried out with the children wearing only underclothes and no shoes. Height and weight were measured and the BMI (weight [kg]/height [m^2^) was calculated. Since height and BMI are age- and gender-related, these parameters were transformed into a standard deviation score (SDS), based upon the established Center for Disease Control normative curves [10]. Overweight and obesity were defined according to Cole et al. [11]. Fractures were confirmed radiographically at the time of injury. Using information about each event documented in the medical record, fractures were classified according to their anatomic site (upper arm/shoulder, elbow/forearm, wrist/hand, hip/thigh, lower leg, and foot) following the WHO International Statistical Classification of Diseases and Related Health Problems [12], and circumstances surrounding the fracture. Children were assigned to a trauma level category based on a modified Landin’s description [13,14] that considers the height of the fall and the landing surface [15,16], the physical activity engaged in, and whether or not any equipment was being used. The study also included a questionnaire assessment by parents regarding socioeconomic factors (parents’ education) and some behavioural issues about the child, such as sports participation in the previous 12 months, and daily hours spent in television viewing. The total daily calcium intake was calculated using a food-frequency questionnaire, specifically established for a pediatric population [17].

### Statistical analysis

All statistical analyses were carried out using the Statistical Package of Social Sciences (SPSS, Windows release 17.0; Chicago, IL, USA). The results are reported as mean ± Standard Deviation (SD), or median and range. The Kolmogorov-Smirnov goodness-of-fit test was applied for determining whether sample data likely derive from a normally distributed population. Variables not normally distributed (BMI-SDS) were logarithmically transformed. However, for clarity of interpretation, results are expressed as untransformed values. The independent sample *t*-test was used to compare the means of continuous variables, while the chi-square test was used for categorical variables (sport participation, TV viewing ≥2 hours/day). A p value < 0.05 was considered significant.

## Results

Demographic characteristics of cases and controls are shown in Table 1. Height, height-SDS, weight, BMI and BMI-SDS were significantly different between the two groups, while no difference was found in parents’ educational level.

**Figure 1:**
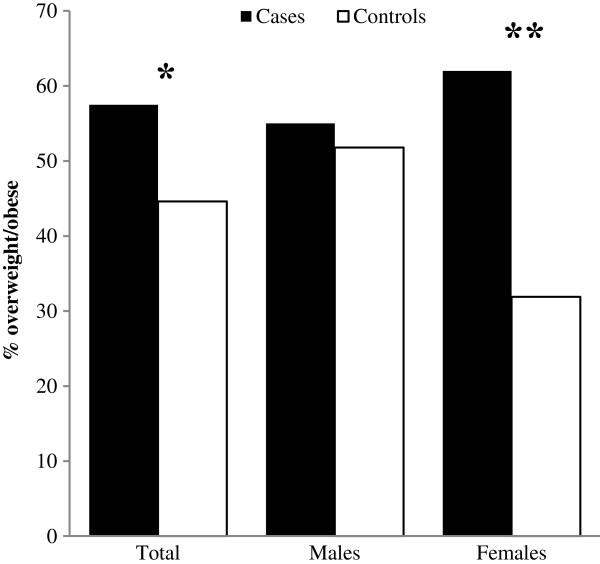
**Prevalence of overweight/obesity in cases and controls, considered as the whole sample or stratified by gender.** The chi-squared test was used to compare the proportions of overweight/obese children between groups. *p = 0.01; **p < 0.001.

Prevalence of overweight/obesity was significantly higher in cases than controls (p = 0.01). However, when gender-specific analyses were run, the greater prevalence of overweight/obesity was confirmed only in girls with fractures (p < 0.001) (Figure 1). Low energy trauma (such as falling to the ground from standing height or bed, playing or sporting injuries) occurred in 319 children (71.1%), while moderate energy trauma (such as falling downstairs, or from a bicycle or while moving on skateboards or rollerblades) in 130 children (28.9%). Prevalence of overweight/obesity did not differ between these two groups (56.7% and 59.2%, respectively).

**Figure 2:**
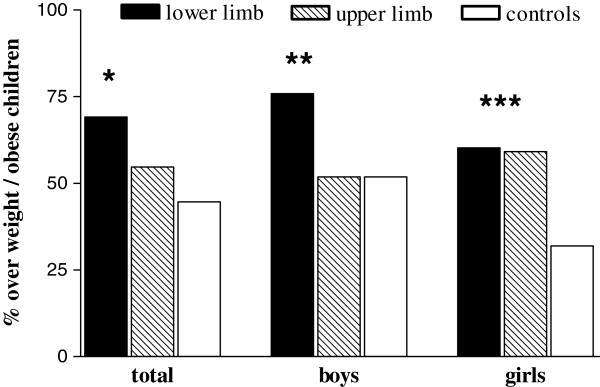
**Prevalence of overweight/obesity in children with lower, or upper limb fractures and controls.** The chi-squared test was used to compare the proportions of overweight/obese children among groups. *p = 0.045 lower vs upper limb; p < 0.002 lower limb vs controls; p < 0.05 upper limb vs controls. **p < 0.01 lower limb vs upper limb; p < 0.02 lower limb vs controls. ***p < 0.004 lower and upper limb vs controls.

Subsequently, we analyzed whether overweight/obesity was specifically associated with the site of fracture. Excluding 43 cases in whom the site was not reported, 351 (86.5%) children reported upper limb fracture and 55 (13.5%) lower limb fracture. Prevalence of overweight/obesity was significantly higher in the lower limb group (69.1%) than the upper limb group (54.7%, p = 0.045); in turn, each group significantly differed from controls (44.6%, p < 0.002 and p < 0.05, respectively). The same comparison was then separately performed by gender. In boys, prevalence of overweight/obesity was 75.8% in the lower limb group and 51.8% in the upper limb group (p < 0.01), while no difference was found between upper and lower limb groups (60.2% and 59.1%, respectively) in girls (Figure 2). Compared to their gender related controls, prevalence of overweight/obesity was higher solely in boys with fracture at the lower limb (p<0.02), and in girls with fractures either at the lower or the upper limb (p= 0.004). Among the various sites of fractures, elbow/forearm fractures were the most prevalent (43.8%), therefore further analyses were performed only in this group. Again a higher prevalence of overweight/obesity with respect to controls was found in girls (61.7% vs 31.9%, p = 0.002), while no difference was found in boys (45.8% vs 51.8%, p 0.398).

Lifestyle behaviours were compared between cases and controls (Table 2). Sports participation as well as TV viewing ≥ 2 hours were higher in cases than controls (sports participation 47.4% vs 33.1%, p = 0.004; TV viewing ≥ 2 hours 57.0% vs 36.9%, p < 0.001). Daily calcium intake did not differ between groups (p= 0.925). In particular, fifty-three fractures (11.8%) occurred from organized sporting activities: 56.6% from soccer, 22.6% from roller skating, 7.5% from gymnastics, 3.4% from basketball, dance or horse riding, and 1.9% from judo. Significant differences in lifestyle behaviours were also found in gender-stratified groups (Table 2).

**Table 2:** Behavioural characteristics in cases and controls as a whole sample or stratified by gender

	**All children**			**Boys**			**Girls**		
	**Controls N= 130**	**Cases N= 449**	**P**	**Controls N= 83**	**Cases N= 292**	**p**	**Controls N=47**	**Cases N= 157**	**p**
Sports participation n (%)	43 (33.1)	213(47.4)	0.004	29 (34.9)	153(52.4)	0.005	14 (29.8)	60(38.2)	0.305
TV ≥ 2 hrs/day n (%)	48(36.9)	256(57.0)	<0.001	35(42.2)	177(60.6)	0.003	13(27.7)	79(50.3)	0.006
Calcium intake mg/day	1137±431	1141±404	0.925	1175±436	1120±402	0.282	1069±419	1178±407	0.113

Data are expressed as number (%) or mean±SD, as appropriate.

The chi-squared test was used to compare proportions (sport participation, TV viewing ≥2 hours/day), while the independent sample *t*-test was used to compare means of daily calcium intake between case/control group.

In order to analyse whether modifiable lifestyle behaviours might have a specific potential role in fracture risk, separate analyses were performed in groups of non-obese and overweight/obese children. Sports participation was higher in children with fractures than fracture free controls (48.7% vs 31.9%, p =0.015) in the normal weight group, while no difference was found (46.1% vs 34.5%, respectively p = 0.107) in the overweight/obese group. Instead TV viewing ≥ 2 hrs was more frequent in children with fractures than fracture free controls in the overweight/obese group (61.5% vs 34.5%, p = 0.015), while no significant difference was found (51.1% vs 38.9%, p = 0.078) in the normal weight group.

## Discussion

The main results of this study confirm a greater prevalence of overweight/obesity in children and adolescents with a recent fracture compared to age and gender matched fracture-free children, but indicate different patterns related to gender and site of fracture. Overweight/obesity rate was increased in girls with fracture either at the upper or the lower limb, while it was increased only in boys reporting a fracture at the lower limb.

While obesity in the adult population has been found to be an independent risk factor of trauma-related morbidity, but not of fractures [5], fractures have been enlisted among orthopaedics complaints of childhood obesity [6,7,18]. In pediatric trauma patients, obese children had a higher incidence of extremity fractures and also a higher risk for certain complications [8]. The reasons for increased numbers of fractures in the obese adolescent population are variably questioned. Reduced bone mass may be a predictor of fracture in children [19], but contradictory results were reported on skeletal mass in overweight/obese children. Since most studies found normal or increased bone mineral content in obese children [20,21], the main conclusion was that obese children have decreased bone mass relative to bone size and body weight [19,22]. It is believed that the increased bone mineral density in obese adolescents may be not sufficient to overcome the significant greater forces that are generated when an overweight child falls.

The assumption of a greater prevalence of fractures in overweight than non-overweight children derives from a retrospective study carried out by Taylor et al. [7] on medical charts of overweight and non-overweight children and adolescents. In their study, Authors did not consider the different sites of fractures or gender related differences. Interestingly, female gender prevailed in their study population of overweight patients compared to non overweight participants.

Apart this study, most Authors analysed the association between weight status and upper limb fractures, in particular distal forearm fractures, which indeed represent the most common fractures in childhood [23]. Our finding of a greater prevalence of overweight/obese subjects among girls with upper limb fractures and in particular with forearm/wrist fractures is confirmed by other studies. Goulding et al. [24] carried out a case–control study about distal forearm fractures in young girls and observed that girls with fractures tended to be heavier than those without, particularly in the 8–10 years age group. As in our study, they excluded fractures from traffic accidents, but their sample included also trauma from severe dynamics (fall > 3 meters) and a high proportion of patients with recurrent fractures. The same authors in a 4 year follow-up study of fractured and fracture-free girls demonstrated that previous fracture, age, total body bone mineral density but also weight were significant factors predicting the risk of new fracture [25]. Skaggs et al. [26] analyzed the skeletal phenotype in low-energy impact fractures of the forearm in girls. At variance with Goulding et al. [24], they could not be able to find any difference in body mass among girls with and without fractures, since their control population was also matched for height and weight. However, they reported that participants were overall 12 kg overweight when compared with age-adjusted normal percentiles for growth. They proposed that the smaller cross-sectional diameter combined with increased body mass and minor trauma created a predisposition to fracture in these patients. With regard to boys, we did not find any significant difference in the prevalence of overweight/obesity between cases and controls when total fractures, upper limb fractures or elbow/forearm fractures were analyzed. In contrast with our results, Goulding et al. [27] showed that high BMI tripled the fracture risk (O.R. 3.47, 95% IC 1.69-7.09) in boys who sustained forearm fractures. Although the discrepancy with our results remains unexplained, the male population studied by Goulding, as in the female study, included trauma from severe dynamics and a high proportion of patients with recurrent fractures.

Instead, we found a greater prevalence of overweight/obesity both in girls and boys sustaining lower limb fracture, in particular foot and tight. As far as we are acknowledged on, several studies reported an increased risk of lower extremity injuries (including fractures, dislocations, sprains/strains) in overweight or obese children [28], while no previous study specifically focused on the association between overweight and lower limb fractures in children. We are only aware of several studies reporting a greater prevalence of ankle fractures in obese adults [29,30]. Although the exact biomechanics of lower extremity injuries in overweight children is largely unknown, the extra weight superimposed to the leg and foot in the overweight subject may explain the increased risk of injuries at the lower extremities. Higher prevalence of abnormal lower extremity alignment has been reported in overweight children, explaining the detrimental effect of obesity to the lower extremity [7,18]. Poor balance, unstable postural sway, altered kinetic characteristics of locomotion in overweight children could be additional risk factors which involve lower extremities [31,32]. The reason why overweight boys may be mainly exposed to the risk of lower limb fracture and not to upper limb fractures is presently difficult to explain, but it could be also viewed in the light of different risk taking behaviours and/or poor intrinsic coordination [33]. Unfortunately we did not assess movement skills and coordination in our population, therefore no final conclusion can be drawn.

There are no data indicating that overweight children fall more frequently than normal weight peers, while it has been only suggested that they fall with more force and in more ackward position [34]. We found that prevalence of overweight/obesity did not differ between groups with low or moderate trauma. In both groups the dynamics was represented by falling, while the difference lied in the height of the fall, the landing surface, the physical activity engaged in, and whether or not any equipment was being used. Our data suggest that the greater overweight rate in children with fractures does represent an inherent vulnerability of the obese child and is not related to the characteristics of the fall itself.

Since lifestyle behaviours have been highly associated with fracture risk in children [1-4,14], the role of physical activity and inactivity was analyzed also in our sample. Sports participation as well as TV viewing ≥ 2 hours were significantly higher in cases than controls. Previous studies reported that higher levels of sports participation increase the risk of fracture in children [1]. This effect, that was independent on the higher bone mineral density and bone size relative to body size determined by the increased volumes of vigorous physical activity [35], was presumably explained through increased exposure to injuries. In our sample 11.8% fractures occurred from organized sporting activities: soccer was the most common cause of injury (56.6%), followed by roller skating (22.6%). Indeed soccer is one of the most popular activities especially among younger boys in our Country, and has a high rate of low-energy trauma, while roller skating, mainly practiced by girls, may be associated with moderate energy trauma [13].

A dose-dependent association between TV, video and computer viewing and wrist/forearm fractures was also reported in children aged 9–16 years [1]. Inactivity may lead to impaired postural balance and reduced muscular strength and coordination,contributing to an increased risk of falling. TV viewing, the most relevant proxy for sedentary lifestyle, has consistently been found associated with childhood obesity [36]. In our study TV viewing ≥ 2 hours was greatly prevalent in overweight/obese children with fractures compared to overweight/obese children without fractures.

Interestingly, more TV viewing was related to smaller gains in bone area and bone mass accounting for race, sex, and height in pre-schoolers, while no effect was found by physical activity measured by accelerometer [37].

Calcium intake is also a strong determinant of mineral accrual in growing children [38,39] and inadequate calcium intake could increase the risk of fracture. Our results did not show any association between dietary intake of calcium and risk of fractures, in agreement with previous reports [35].

The strength of the present study is based on the fact that we provided objective measurements of height and weight (hence, the accurate estimate of BMI) rather than self-reported height and weight, which is a potential source of error caused by reporting bias. Moreover all fractures were confirmed radiographically and the dynamics of trauma was accurately ascertained. Our study also has a limitation in the lower sample size of hospitalized controls. This was due to the difficulty of enrolling the control population, mainly girls, from the same setting (hospital) of the case population. Notwithstanding this limitation, the prevalence of overweight/obesity observed in our control population (boys 51.8%, girls 31.9%) reflects the overweight/obese prevalence indicated by the Health Behaviour in School Children survey performed in 2009–2010 in our region (boys 45%, girls 28% at 11 years of age) [40,41], according to the definition used by the International Obesity Task Force [11].

## Conclusions

In conclusion, this study provides a comprehensive overview of the complex relationship between overweight/obesity and pattern of fractures in the pediatric population. Higher inactivity seems to characterize overweight children reporting fractures. Research exploring the effects of obesity on the risk of falling and the protective responses employed while falling will provide a better understanding of underlying mechanisms.

## Competing interests

The authors declare that they have no competing interests.

## Authors’ contribution

GV and GL provided substantial contributions to the conception and design of the study, definition of the objectives, development of the questionnaire, and analysis and interpretation of data; they revised the paper critically for important intellectual content. FG and VDO provided substantial contributions to background analysis and literature research, analysis and interpretation of data. CM provided substantial contributions to acquisition of data, parents’ interviews, development of the database, and uploading of the data in a dedicated software. PG, AT and ER provided substantial contributions to the enrolment of cases and controls, design of the study, definition of the objectives; they revised the paper critically for important intellectual content. All authors were involved in writing the paper and had final approval of the submitted and published versions.

## Pre-publication history

The pre-publication history for this paper can be accessed here:

http://www.biomedcentral.com/1471-2431/12/166/prepub
